# Contrasting drivers of reproductive ageing in albatrosses

**DOI:** 10.1111/1365-2656.12712

**Published:** 2017-07-17

**Authors:** Hannah Froy, Sue Lewis, Daniel H. Nussey, Andrew G. Wood, Richard A. Phillips

**Affiliations:** ^1^ Institute of Evolutionary Biology University of Edinburgh Edinburgh UK; ^2^ British Antarctic Survey Natural Environment Research Council Cambridge UK; ^3^ Centre for Ecology and Hydrology Bush Estate Penicuik UK

**Keywords:** Bird Island South Georgia, black‐browed albatross *Thalassarche melanophris*, grey‐headed albatross *Thalassarche chrysostoma*, life‐history trade‐off, selective disappearance, senescence, terminal effect, wandering albatross *Diomedea exulans*

## Abstract

1. Age‐related variation in reproductive performance is ubiquitous in wild vertebrate populations and has important consequences for population and evolutionary dynamics.
2. The ageing trajectory is shaped by both within‐individual processes, such as improvement and senescence, and the among‐individual effects of selective appearance and disappearance. To date, few studies have compared the role of these different drivers among species or populations.
3. In this study, we use nearly 40 years of longitudinal monitoring data to contrast the within‐ and among‐individual processes contributing to the reproductive ageing patterns in three albatross species (two biennial and one annual breeder) and test whether these can be explained by differences in life histories.
4. Early‐life performance in all species increased with age and was predominantly influenced by within‐individual improvements. However, reproductive senescence was detected in only two of the species. In the species exhibiting senescent declines, we also detected a terminal improvement in breeding success. This is suggestive of a trade‐off between reproduction and survival, which was supported by evidence of selective disappearance of good breeders.
5. We demonstrate that comparisons of closely related species which differ in specific aspects of their life history can shed light on the ecological and evolutionary forces shaping variation in ageing patterns.

## 1 INTRODUCTION

Age‐related variation in reproductive performance is a ubiquitous feature of wild vertebrate populations (Clutton‐Brock, 1988). At the population level, this variation is underpinned by complex interactions between processes at the within‐ and among‐individual levels (Forslund & Pärt, 1995; Nussey, Coulson, Festa‐Bianchet, & Gaillard, 2008; van de Pol & Verhulst, 2006). Within‐individual changes with age commonly include increasing performance through early adulthood—driven by the benefits of greater foraging efficiency (Daunt, Wanless, Harris, Money, & Monaghan, 2007), competitive ability (Forslund & Pärt, 1995) or mate familiarity (Black & Hulme, 1996)—followed by a plateau and then decline, as the physiological deterioration associated with senescence takes hold (Brunet‐Rossinni & Austad, 2006; Rose, 1991). Recent studies suggest that in some species, within‐individual ageing may be discontinuous, i.e. characterized by abrupt, stepwise changes (Bouwhuis, Sheldon, Verhulst, & Charmantier, 2009). For example, much lower performance in first‐time breeders compared to other young adults, and low performance at the final breeding attempt (BA), presumably due to terminal declines in physiological condition, have been observed (Coulson & Fairweather, 2001; Rattiste, 2004). Among‐individual processes are also important drivers of population‐level changes in breeding performance with age (Cam & Monnat, 2000; McCleery, Perrins, Sheldon, & Charmantier, 2008; Reid et al., 2010). For instance, lower quality individuals may show delayed onset of reproduction, and their selective appearance in the breeding population could mask within‐individual improvements with age in early adulthood (van de Pol & Verhulst, 2006). Poor‐quality animals may also have shorter life spans and become increasingly under‐represented in older age classes, potentially masking within‐individual senescent declines in longer‐lived animals (Aubry, Koons, Monnat, & Cam, 2009; Vaupel, Manton, & Stallard, 1979). Over the last few decades, researchers working with longitudinal data have repeatedly demonstrated the importance of these different within‐ and among‐individual processes in wild vertebrate populations (Aubry et al., 2009; Hayward et al., 2013; Rebke, Coulson, Becker, & Vaupel, 2010; Reed et al., 2008; Reid et al., 2010; Robinson, Mar, & Lummaa, 2012; Zhang, Vedder, Becker, & Bouwhuis, 2015). However, direct comparisons of the relative importance of these processes across species and populations remain rare (Balbontín et al., 2012; Bouwhuis, Charmantier, Verhulst, & Sheldon, 2010; Holand et al., 2016; Nussey et al., 2011), and our knowledge of the ecological and evolutionary forces responsible for observed differences is currently limited.

Trade‐offs between current and future reproduction are key in shaping age‐specific performance (Williams, 1966). Life‐history theory states that individuals must allocate limited resources between the competing functions of growth, maintenance and survival on one hand and reproduction on the other (Stearns, 1992). These trade‐offs will influence both the ageing trajectory and the underlying processes. For example, individuals that invest heavily in reproduction in early life may experience a cost in terms of senescence, exhibiting earlier onset and more rapid declines in performance in later life (Nussey, Kruuk, Donald, Fowlie, & Clutton‐Brock, 2006; Reed et al., 2008). There could also be a survival cost (Boonekamp, Salomons, Bouwhuis, Dijkstra, & Verhulst, 2014; Hayward, Mar, Lahdenperä, & Lummaa, 2014), which would result in their selective disappearance from the population and contribute to age‐related declines in breeding success at the population level (Reid et al., 2010). Evidence of these trade‐offs may be apparent at multiple scales of observation—among individuals, populations and species. Broad‐scale species comparisons have highlighted associations between the onset of, and rate of senescence, and life‐history traits such as age at first reproduction, fecundity and generation time (Jones et al., 2008; Péron, Gimenez, Charmantier, Gaillard, & Crochet, 2010; Ricklefs, 2010). These studies suggest that senescence rates can be predicted by the alignment of species on the fast–slow life‐history continuum and provide a framework for further species comparison. Comparisons of closely related species, which exploit similar ecological niches but differ in specific aspects of their life‐histories, may provide valuable insights into the drivers of different ageing trajectories. However, few studies have examined age‐related variation in reproductive performance among closely related species (though see Berman, Gaillard, & Weimerskirch, 2009; Loison, Festa‐Bianchet, Gaillard, Jorgenson, & Jullien, 1999), and fewer still have decomposed these patterns to better understand the underlying drivers (Nussey et al., 2011).

Here, we examine age‐specific breeding success in three extremely long‐lived species of seabird and decompose the population‐level patterns to better understand the relative importance of within‐ and between‐individual processes in shaping the ageing trajectory in each species. We used data collected from long‐term, individual‐based studies of wandering (*Diomedea exulans*), black‐browed (*Thalassarche melanophris*) and grey‐headed (*Thalassarche chrysostoma*) albatrosses at Bird Island, South Georgia. These species are extremes on the fast–slow continuum, characterized by high adult survival, late sexual maturity and low reproductive rate. We, therefore, predict that, as observed in most other long‐lived vertebrates, all species will show within‐individual improvements during early life, followed by reproductive senescence. However, breeding frequency differs between these three species; wandering and grey‐headed albatross generally exhibit a biennial breeding tactic, breeding every other year if successful, whereas the black‐browed albatross breeds annually. Assuming this higher breeding frequency reflects a greater investment in reproduction in the black‐browed albatross, life‐history theory predicts that this species will recruit at a younger age, senesce earlier and faster, and show stronger selective disappearance associated with high average breeding success compared with the two biennial species.

## 2 MATERIALS AND METHODS

### 2.1 Study system

South Georgia is a globally important breeding site that holds 10%–50% of the world populations of black‐browed, grey‐headed and wandering albatrosses (ACAP, 2014a, 2014b, 2014c), hereafter referred to as BBAs, GHAs and WAs, respectively. Average adult body mass of WA is *c*. 9 kg, considerably larger than BBA and GHA which both weigh *c*. 3.5 kg. All three species are sexually size dimorphic, with males 15%–20% heavier than females (Phillips, Silk, Phalan, Catry, & Croxall, 2004; Weimerskirch, 1992). WAs have a very wide foraging distribution and feed predominantly on squid and fish (Xavier et al., 2004). BBAs feed predominantly in shelf and shelf‐slope waters, whereas GHAs are more oceanic, targeting deep water and frontal zones (Phillips et al., 2004). At South Georgia, 30%–40% of the diet of GHA and BBA consists of Antarctic krill *Euphausia superba*, with the remainder mainly squid or fish, respectively (Xavier, Croxall, & Reid, 2003).

**Figure 1:**
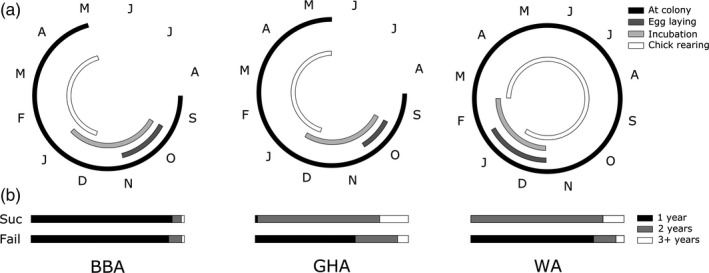
The seasonal cycle of three species of albatrosses: the black‐browed albatross (BBA); grey‐headed albatross (GHA) and wandering albatross (WA). (a) The annual breeding cycle, with lines indicating the periods typically spent: at the colony; egg laying; incubating and rearing chicks. Letters indicate months of the year. (b) The typical gap taken between successive breeding attempts for each species. Bars indicate the proportion of successful or failed breeders that return to breed after 1, 2 or 3+ years (from Croxall et al., 1990; Prince et al., 1994)

Albatrosses lay a single egg each BA, and both parents contribute to incubation and provisioning of the chick until fledging. The breeding cycle is the longest for WA, which takes an entire year to successfully raise a chick (Figure 1a), and lasts 6–7 months for BBA and GHA which do not feed the chick over the austral winter. WA and GHA typically exhibit a biennial tactic; however, unsuccessful breeders may breed the following year, although this is dependent on the timing of failure as those that fail late in the season are likely to defer (Ryan, Phillips, Nel, & Wood, 2007; Figure 1b). BBAs are typically annual breeders regardless of the outcome of their previous BA. However, all three species take a variable number of years off, and the proportions differ between years (Prince, Rothery, Croxall, & Wood, 1994). In WA, *c*. 30%–35% of birds defer breeding beyond the expected year (Croxall, Prince, Rothery, & Wood, 1998).

Data used in this analysis were collected between the 1975/1976 and 2012/2013 breeding seasons as part of a long‐term study at Bird Island, South Georgia (54°00′S, 38°03′W). These are referred to as years 1976–2013 from here, denoting the year in which the chick fledges. Albatross chicks of all three species have been ringed annually on the island since the 1970s (although intermittent ringing occurred earlier), and consequently many of the adult breeders are of known age (see Data S1a for further details of sample sizes and ringing schedules). Colonies were visited daily during the pre‐laying, hatching and fledging periods, and at least weekly during incubation and chick rearing. Breeding success was determined for each nesting attempt as a binary trait reflecting whether the chick fledged.

### 2.2 Data analysis

Mean annual breeding success was compared among species. We estimated mean age at first and last reproduction for both sexes of each species, and the difference was considered to be the mean reproductive life span. Given the variation in past ringing schedule and study duration among species, we included only individuals that were ringed and observed over specific time intervals to ensure our estimates were broadly comparable (intervals kept consistent across species; see Data S1b).

The relationships between age and breeding success were modelled separately for each species using generalized linear mixed‐effects models (GLMMs) with a binomial error distribution and logit link function (Zuur, Ieno, Walker, Saveliev, & Smith, 2009). All analyses were performed using the lme4 statistical package in the program R (version 3.2.2).

#### 2.2.1 Age‐related variation in breeding success

Age‐related variation in breeding success was initially modelled without accounting for the effects of selective appearance and disappearance, hereafter referred to as the population‐level analysis. Initially, the sexes were modelled separately for each species because pairs typically remain together for most of their lives, and male and female age is highly correlated within pairs (see Results). To avoid small sample sizes at the extremes of the age range unduly influencing results, we excluded observations where the number of observations per age was <5 (results remain unchanged if we instead grouped ages where the number of observations was <10). All models included individual as a random effect to account for the non‐independence of observations and year as a random effect to account for annual variation in environmental conditions.

Model selection was performed using Akaike information criterion (AIC) where the best model is taken to be that with the lowest AIC value (Burnham & Anderson, 2002). When model selection resulted in multiple models with an AIC difference of less than two units (∆AIC < 2) compared to the model with the lowest AIC, a model averaging approach was used since models where ∆AIC < 2 are not considered to be meaningfully different (Burnham & Anderson, 2002). These models comprised the top model set (Grueber, Nakagawa, Laws, & Jamieson, 2011), and predictions from these models were weighted by AIC weights (ω) and summed to give predictions of the average model using the R package muMin (Barton, 2013). The model‐average estimates were then compared visually with the best model.

The shape of the age function for each species and sex was determined by comparing a variety of candidate models. Null models included year and individual, but no age term. Linear and quadratic age functions were compared with threshold models with a range of breakpoints (following Berman et al., 2009). These piecewise regression models had a single or double threshold, between which the slopes were allowed to vary independently. The tested thresholds depended on the age range of each species (see Data S1c). Where the best model included a threshold term for age, confidence intervals were placed around the threshold value at the 95% limit (following Ulm & Cox, 1989). Model predictions ± standard errors were generated using the *predictSE* function in the package AICcmodavg (Mazerolle, 2016), not accounting for random effects.

The explanatory power of male and female age was then compared for each species using a subset of data for which the age of both parents was known. The best single threshold age function for both males and females were included in a model of breeding success for each species. The male and female terms were dropped in turn, and the change in AIC score examined. The sex that resulted in the greater increase in AIC score was considered to explain more variation in the data.

#### 2.2.2 Factors underpinning variation in breeding success during early and late adulthood

The population‐level analyses indicated that breeding success increased in early adulthood, and then declined or plateaued following a peak between the mid‐teens and mid‐twenties (see Results). In the next set of analyses, these observed patterns were decomposed to reveal the factors underpinning age‐related variation in breeding success. Each dataset was divided into early and late adulthood based on the population‐level model selection for each species and sex. The cut‐off indicated by the best single threshold model was used in each case, except for female WAs where a cut‐off of 18 years was used to ensure all birds had started breeding during early adulthood (following Froy, Phillips, Wood, Nussey, & Lewis, 2013). Individual and year were included as a random effects, and analyses were performed separately for males and females.

The within‐group centring approach (van de Pol & Verhulst, 2006) was used to quantify the relative contributions of the within‐individual and between‐individual effects to the observed age patterns. Two GLMMs were fitted for each species and sex, modelling breeding success in early and late adulthood, respectively. Ages at first or last reproduction were included as fixed covariates in the models in addition to the age term. The parameter estimates for these terms represent the relative contributions of selective appearance and disappearance of phenotypically different individuals from the population. The age term was expressed as years since first BA or years before death, along with age at first or last reproduction, respectively. The parameter estimates for these terms reflect the contribution of within‐individual changes in breeding success to the population‐level average.

In early adulthood (but not late adulthood as all individuals were by then established breeders), it was important to account for the effects of selective appearance in the breeding population, and so the analyses included data from individuals for which age at first measurement reflected the age at first reproduction. In the analyses of late adulthood, only data for individuals for which age at last measurement reflected age at last reproduction were included, allowing us to account for the effects of selective disappearance. This included individuals that did not return to the colony for 5 years for WA and GHA and 4 years for BBA; these were assumed to be dead since established breeders show extremely high site fidelity and very few take longer than this between BAs (<1% of observations in each dataset). Note that we do not know the exact age at death for these birds since it occurs at sea, but we use disappearance from the breeding colony as a proxy.

A binary factor for first BA was included in the early adulthood models to examine how much of the within‐individual increase in breeding success could be attributed to poor performance by first‐time breeders (first BA = 1; subsequent BA = 0). Similarly, a binary factor for final BA was included in each late adulthood model to test for terminal effects (Bouwhuis et al., 2009), comparing the breeding success of individuals on their final BA with previous attempts (last BA = 1; previous BA = 0).

Finally, to check whether the differences among species were detectable, we ran models combining all three species and testing two‐way interactions between species and each of the three age terms (see Data S1d for further details). We also included additional terms in our main late adulthood models to check that our results were independent of these effects. To account for potential negative effects of partner change, we included this as a binary factor in our models (first BA following a partner change = 1; other BA = 0). To check that the effects were independent of the variable time gap between breeding events and its dependence on the previous outcome, we included a covariate for the number of years since the bird last bred, and a binary factor which reflected the outcome of the previous BA (previous BA successful = 1; unsuccessful = 0), and the interaction between these terms.

## 3 RESULTS

Mean annual breeding success varied between species and was lowest in BBA (0.317 ± 0.035), intermediate in GHA (0.403 ± 0.034), and highest and least variable in WA (0.631 ± 0.022) (Figure S1). WA were more likely to fail during the incubation than the chick‐rearing period, whereas the reverse was true for BBA and GHA (proportion of failures during incubation: BBA 0.42; GHA 0.44; WA 0.56). Mean annual breeding success was most closely correlated between GHA and BBA (*r* = .508, *p* = .001), marginally correlated between WA and BBA (*r* = .315, *p* = .084), and showed no detectable relationship between GHA and WA (*r* = −.071, *p* = .703).

Reproductive life span differed among species, reflecting differences in mean age at first and last reproduction (Table 1). Generally, GHA reached sexual maturity later, but had a longer reproductive life span than the other species due to a higher age at last reproduction. Within pairs, mean female and male age was correlated for all species (BBA: *r* = .417, *n* = 185, *p* < .001; GHA: *r* = .512, *n* = 208, *p* < .001; WA: *r* = .744, *n* = 456, *p* < .001).

**Table 1:** Age at first reproduction, age at last reproduction and estimated reproductive life span for three species of albatrosses. Ages are the mean ± standard error. Reproductive life span is calculated as the difference between mean age at first and last reproduction

		*n*	Age at first repro	*n*	Age at last repro	Repro lifespan
BBA	Males	170	9.40 ± 0.13	155	17.89 ± 0.61	8.49
	Females	90	11.78 ± 0.30	82	20.87 ± 0.86	9.09
GHA	Males	157	13.47 ± 0.20	180	27.54 ± 0.67	14.07
	Females	125	13.33 ± 0.19	161	28.63 ± 0.67	15.3
WA	Males	331	10.21 ± 0.13	279	21.33 ± 0.55	11.12
	Females	339	9.73 ± 0.13	276	18.99 ± 0.58	9.26

### 3.1 Population‐level ageing patterns

**Figure 2:**
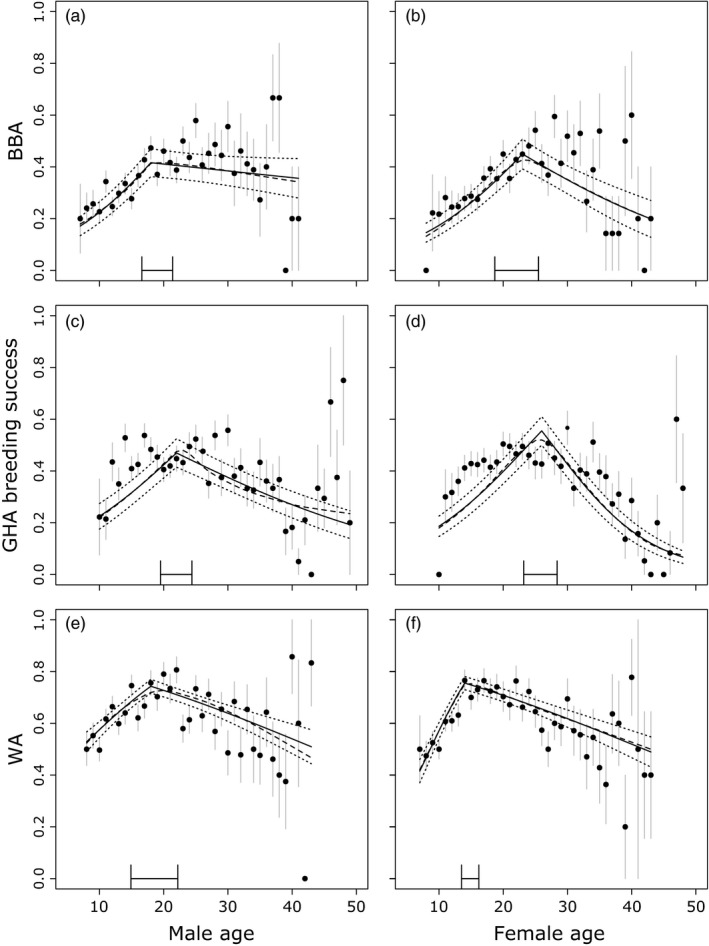
Relationships between age and breeding success. Points and grey bars show mean breeding success for each age, ± standard error. The solid black lines show the ageing pattern predicted by the best single threshold model (Table S2), with dotted lines showing standard errors around this average prediction. The dashed lines show the ageing pattern predicted by the average model. Plots are for the following: (a) male black‐browed albatross (BBA) *n* = 2,273; (b) female BBA *n* = 1,521; (c) male grey‐headed albatross (GHA) *n* = 2,267; (d) female grey‐headed albatross (GHA) *n* = 2,225; (e) male wandering albatross (WA) *n* = 2,255; (f) female WA *n* = 2,260. The bar above the *x*‐axis reflects the estimated 95% confidence intervals around each threshold

All species showed age‐related variation in reproductive success, with the best age function model explaining more variation in the data than the null model including year and individual identity (∆AIC of the null model compared to the model with the best age function: BBA: males ∆AIC = +33.27, females ∆AIC = +20.17; GHA: males ∆AIC = +16.05, females ∆AIC = +59.98; WA: males ∆AIC = +20.27, females ∆AIC = +44.74). In all cases, after model selection, there remained a number of equivalent models that explained the relationship between age and breeding success (Table S1). A single threshold age function had the lowest AIC score for male and female BBA (18 and 23 years, respectively), male and female WA (18 and 14 years, respectively) and female GHA (26 years). For GHA males, the best single threshold age function was within ∆AIC = 0.23 of the best overall model (22 years; Table S1). Averaging across the top model set in each case resulted in an age function that was almost identical to the best single threshold model (Figure 2). Therefore, we proceeded with the best single threshold model in each case (Figure 2; Table S2).

Overall, both sexes in all species showed an increase in breeding success until the mid‐teens to mid‐twenties and then a decline or plateau at the population level. The best threshold value varied between the species and sexes, although the 95% confidence intervals broadly overlapped (Figure 2; Table S2). All sexes and species showed increases in breeding success in early life at the population level (Table S2). However, the slopes after the threshold varied; although all were negative, the slope in BBA males was not statistically different from zero.

In GHA and WA, female age explained considerably more variation in the data than male age (Table S3). Dropping the female age term from a model containing both female and male age functions resulted in a substantial increase in AIC score (GHA: ∆AIC = +11.39; WA: ∆AIC = +16.60), whereas dropping the male term had a negligible effect on AIC score (Table S3). In addition, the parameter estimates for the female terms remained statistically different from zero with both age functions in the models for these two species, whereas the male age terms were not (Table S3). The opposite was true for BBA, where male age explained more variation in breeding success (∆AIC = +7.27 when the male term was dropped; Table S3). Again, the increase in breeding success associated with male age before the threshold remained detectable, whereas both female terms were not statistically different from zero.

### 3.2 Factors underpinning variation in breeding success

The effect of years since first breeding was positive in all species, indicating that breeding success tended to improve progressively within individuals during early adulthood (Table 2a). This was detectable in all cases except for WA males. The analysis testing interactions among species and the age terms indicated that the rate of improvement was faster in BBA (Table S4; see Data S1e). Both males and females of all species tended to perform poorly on their first BA. This was the most important factor explaining breeding success in early life for male WA (Table 2a), indicating that most of the within‐individual improvement occurred on the second BA and not thereafter. The relationship between age at first reproduction and breeding success was generally positive, but only detectable in female BBA, indicating that individuals who delayed the onset of reproduction performed better in early life than those that began breeding earlier (Table 2a). However, the analysis of all species together suggested an absence of difference among species, with all females showing a positive effect of selective appearance on breeding success (Table S4).

**Table 2:** Estimated fixed effects (estimate and standard error [*SE*]) from GLMMs of breeding success for birds during (a) early adulthood and (b) late adulthood. All models included year and bird ID as random effects. The effect of removing each parameter on the model AIC is shown (∆AIC). Positive ∆AIC values indicate that the term improved model fit, and those parameters whose removal increased AIC by >2 are highlighted in bold

(a)		*n*	Estimate	*SE*	∆AIC	Estimate	*SE*	∆AIC	Estimate	*SE*	∆AIC
			Years since first bred			Age at first repro			First breeding attempt		
BBA	Males	953	**0.204**	**0.040**	**24.832**	−0.052	0.069	−1.438	−0.007	0.277	−1.999
	Females	588	**0.190**	**0.038**	**25.583**	**0.137**	**0.057**	**3.809**	−0.501	0.365	−0.048
GHA	Males	612	**0.153**	**0.049**	**8.021**	0.058	0.053	−0.844	−0.375	0.326	−0.699
	Females	670	**0.095**	**0.033**	**6.282**	0.027	0.062	−1.819	−0.625	0.330	1.615
WA	Males	1,258	0.010	0.034	−1.914	0.020	0.046	−1.807	−**0.815**	**0.200**	**15.160**
	Females	1,327	**0.114**	**0.035**	**8.638**	0.073	0.050	0.101	−**0.441**	**0.197**	**3.078**
**(b)**		***n***	**Years before death**			**Age at last repro**			**Last breeding attempt**		
BBA	Males	455	0.067	0.036	1.412	−0.030	0.023	−0.287	−0.039	0.348	−1.987
	Females	182	0.033	0.072	−1.795	−0.106	0.062	0.744	−0.178	0.613	−1.915
GHA	Males	838	**0.091**	**0.028**	**8.839**	−**0.066**	**0.024**	**5.555**	**0.786**	**0.271**	**6.300**
	Females	630	**0.209**	**0.046**	**20.332**	−**0.126**	**0.040**	**8.501**	**1.016**	**0.354**	**6.380**
WA	Males	711	**0.078**	**0.026**	**7.463**	−0.031	0.017	1.273	**0.543**	**0.234**	**3.378**
	Females	575	**0.069**	**0.025**	**5.571**	−0.029	0.017	0.751	**0.701**	**0.258**	**5.485**

In late adulthood, the effect of years before death was consistently positive across all species and sexes, indicating that breeding success declined as individuals approached death. This was detectable in both male and female GHA and WA, but not in BBA (Table 2b). Combining data from all three species suggested that the rate of decline did not differ among males, but female GHA showed a more rapid senescent decline in performance (Table S5). The effect of age at last reproduction was also consistent; both sexes in all species showed a negative relationship between age at last reproduction and average breeding success. This indicates selective disappearance of high‐performing individuals; those with greater average breeding success in late adulthood had reduced life span. This effect was only statistically significant for male and female GHA, indicating that some of the population‐level declines in breeding success in this species are likely to be driven by selective disappearance (Table 2b). The analysis of all three species suggested that the effect was weaker in female WA (Table S5). The effect of last BA on breeding success differed between the species in the separate analyses and was positive in GHA and WA, indicating an increase in breeding success on the final BA which contrasts with the general decline as individuals approached death (Table 2b). All late adulthood effects were independent of partner change (Table S6), as well as the number of years since the last BA, the outcome of that previous attempt and the interaction between these terms (Table S7).

## 4 DISCUSSION

Our study found evidence for considerable variation in reproductive performance with age in the three confamilial species of long‐lived seabird and marked differences in the ageing patterns. As predicted, there was an increase in breeding success during early adulthood in all species, driven predominantly by improvements in individual performance. There was evidence for trade‐offs between reproduction and survival in GHA, with the selective disappearance of good breeders and a terminal increase in breeding success. In contrast with our expectations, reproductive senescence was detectable in GHA and WA but not BBA.

GHA are the longest lived of the three species in our dataset. Although they reach sexual maturity later, as indicated by the high age at first reproduction, GHA have the highest mean age at last reproduction and consequently the longest reproductive life span (Table 1). It should be noted that albatross populations are declining as a consequence of incidental mortality (bycatch) in fisheries, but as reliable data on bycatch rates are not available for many fleets, seasons and areas (Phillips et al., 2016), the extent to which bycatch affects the mean reproductive life span in each species is unknown. However, removal of a small percentage of individuals from the sample through bycatch is unlikely to cause biases in the ageing patterns that we observed. Life‐history theory predicts a trade‐off between reproduction and survival, due to the allocation of limited resources between fitness traits (Stearns, 1992). GHA are typically biennial breeders and will skip the following season if they fledge a chick; however, a small minority of successful breeders attempt to breed annually, particularly if environmental conditions are favourable (Ryan et al., 2007). Less frequent attempts at breeding could be indicative of a lower relative allocation in reproduction compared to the congeneric BBA, which usually breeds annually (Prince et al., 1994). GHA at South Georgia also had relatively low annual breeding success (*c*. 40%), compared to WA, another biennially breeding species that successfully fledges its chick on around two‐thirds of attempts (Figure S1). Although the ultimate drivers are uncertain, these differences are suggestive of variation in a life‐history trade‐off between the species, with lower reproductive allocation in the GHA and an increased allocation of resources towards survival and maintenance, leading to an extended reproductive life span.

Male age explained more variation in breeding success than female age in BBA pairs, while female age was more important in the other two species (Table S3). This could be because male BBA deliver more food than females to the chick (Huin, Prince, & Briggs, 2000). Although males are heavier (Phillips et al., 2004), provisioning capability (meal mass and feeding frequency) in albatrosses and other seabirds does not scale isometrically with body mass (Phillips & Hamer, 2000). The burden of maintaining a higher feeding rate may, therefore, incur a greater cost for males, and if so, their age and competency could be more important in determining reproductive success. In contrast to BBA, male and female GHA provision the chick equally (Huin et al., 2000). As males are also heavier in this species, equal food provisioning is likely to be more costly for females, which could explain why female age accounts for more variation in breeding success. In WA, as in BBA, males deliver more food to the chick (Berrow, Humpidge, & Croxall, 2000). However, the rate of failure is higher during incubation than chick rearing for WA, whereas the reverse is true in BBA and GHA, which fail more frequently during chick rearing. Studies have shown that although male and female WA lose similar proportions of their body mass during incubation shifts, males regain mass more quickly while at sea (Croxall & Ricketts, 1983; Weimerskirch, 1995). Therefore, female fasting capacity may be more important during this critical period for WA, and thus female age explains more variation in breeding success.

The population‐level analysis revealed increases in breeding success during early adulthood in BBA, GHA and WA, and decomposing these patterns revealed that they were predominantly driven by within‐individual improvements in performance (Table 2a). This is consistent with the proposed benefits of development, experience and learning for birds in general (Forslund & Pärt, 1995). In BBA, GHA and female WA, progressive improvements were evident throughout early adulthood (Table 2a). Selective appearance was detected only in BBA females, with birds tending to do better if they delayed reproduction. However, in nearly all cases, the direction of the effect was consistent, suggesting that selective appearance of good breeders may contribute somewhat to the population‐level increase (Tables 2a and S4). Albatrosses take many years to reach sexual maturity, and thus are expected to be proficient foragers at the time of recruitment. However, new skills may be required to successfully raise a chick, such as coordinating with the mate, and adapting to the constraints imposed by central place foraging, resulting in improvements that can only be gained with experience of breeding per se (Croxall, Rothery, & Crisp, 1992; Weimerskirch, 1992). An additional analysis showed that the rate of improvement was more rapid in BBA males and females when compared to the other species (Table S4). This could, perhaps, reflect the younger recruitment age and annual breeding tactic in BBA which provides more opportunities to gain experience per unit time. The poor performance of first‐time breeders was most apparent in WA, where perhaps coordination between mates of incubation shifts, which can be particularly long in this species, plays a more important role in determining the breeding outcome.

Reproductive performance appeared to peak latest in GHA females (Figure 2), which is consistent with a later onset of senescence predicted for this longer‐lived species (Jones et al., 2008). Following peak performance, there was evidence for reproductive senescence in late adulthood in WA (as seen in Pardo, Barbraud, & Weimerskirch, 2013) and GHA but, surprisingly, not in BBA (Table 2b). This contrasts with predictions from life‐history theory, since the rate of senescence is expected to be more rapid in the species with shorter generation time and lower age at first reproduction (cf. southern fulmar *Fulmarus glacialoides* with the longer‐lived snow petrel *Pagodroma nivea*; Berman et al., 2009). Our analyses combining all three species show that although GHA females showed the most rapid senescence, rates of senescence did not differ significantly between WA and BBA (Table S5). The lack of a detectable senescent decline in BBA in the separate analyses is likely due to a lack of power, rather than evidence against reproductive senescence per se. The more rapid senescent declines detected in female GHA could be attributable to differences in ecological conditions experienced by this pelagic species, as breeding success of the population at South Georgia is considerably lower than the same species in the Indian Ocean (Ryan et al., 2007), and senescent declines may be exacerbated or more readily detectable when conditions are harsh (Reznick, Nunney, & Tessier, 2000). The lack of an observable decline in breeding success in BBA may be related to their lower mean and more variable annual productivity at Bird Island, which could be attributed partly to stochasticity in levels of egg and chick predation by other seabirds (Forster & Phillips, 2009) and in the abundance of their main food source, Antarctic krill (Croxall, Reid, & Prince, 1999). Importantly, other populations of BBA that do not rely on this prey source tend to have higher and more consistent breeding success (Nevoux, Forcada, Barbraud, Croxall, & Weimerskirch, 2010), and they show clear signs of senescent declines in reproductive performance (Pardo, Barbraud, Authier, & Weimerskirch, 2013). These deviations from life‐history theory predictions suggest that although the fast–slow continuum is a very useful framework for understanding senescence rates, more detailed ecological knowledge may be needed to explain observed differences among species and populations.

The effect of age at last reproduction was negative in all three species, although this only explained part of the observed variation in the breeding success of GHA (Table 2b). This indicates that birds with higher than average reproductive success in late adulthood tend to have lower survival rates. Most studies that examine the effects of selective disappearance in wild populations find the opposite effect, i.e. quality effects predominate, and individuals with higher reproductive performance also tend to be longer lived (Bouwhuis et al., 2009; Dugdale, Pope, Newman, MacDonald, & Burke, 2011; McCleery et al., 2008; Nussey et al., 2006). This selective disappearance of poor‐quality individuals from successive age classes can mask senescent declines in the performance of longer‐lived animals (Nussey et al., 2008). The negative relationship between age at last reproduction and breeding success in this study indicates that the population‐level declines observed in the GHA are partly driven by compositional effects. This is indicative of a trade‐off between reproduction and survival, which is not commonly observed in long‐lived species (Hamel et al., 2010). This is partly because individuals may only choose to breed if they can afford to do so without suffering costs (Reid et al., 2010), and also because survival costs of reproduction are less easily detected than reproductive costs, since variance in breeding success is higher than variance in survival in species with slow life histories (Hamel et al., 2010). This result also corroborates the suggestion that grey‐headed albatrosses have greater longevity because of their low reproductive investment; when they do invest heavily in reproduction, they incur a survival cost.

The results suggest that such a trade‐off is evident in both the short and long term, since GHAs also show an increase in breeding success on the final attempt before they disappear from the population (Table 2b). This terminal improvement was also documented in a previous study of WA (Froy et al., 2013), a pattern which is consistent with both predictions of terminal investment and a high cost of reproduction. This effect was not detected in the separate analyses of BBA, which is the predominantly annual breeder; however, this difference among species was not statistically significant (Table S5). In GHA, the nature of the biennial breeding cycle means that successful breeders may be subject to a longer mortality period than failed breeders. Although WA are also biennial breeders, their extremely long breeding cycle takes an entire year (Figure 1). Therefore, successful breeders will have already survived for a year while raising their chick, and the length of the subsequent sabbatical, therefore, matches broadly with the duration of the non‐breeding period of a failed breeder (Croxall, Rothery, Pickering, & Prince, 1990). However, because the breeding season of GHA does not last a full year, a bird defers breeding for 16 months following a successful BA. It is plausible that this drives the increase in breeding success on the final attempt (in effect, an increased likelihood of mortality following this terminal success). This is a complex issue, since the intervals between BAs are variable for all the species. However, it should be noted that the observation of increased performance on the final BA is extremely unusual in a wild system, and in stark contrast to the terminal declines observed in other species (Hammers, Richardson, Burke, & Komdeur, 2012; Nussey et al., 2011), including some seabirds (Coulson & Fairweather, 2001; Rattiste, 2004).

Overall, this study demonstrates extensive age‐related variation in reproductive performance in three long‐lived species of seabird. There were many commonalities in terms of age‐related variation, which is to be expected for such closely related and ecologically similar species. In particular, the ageing patterns and drivers in early life were broadly comparable, with the importance of within‐individual improvement evident in all cases. There were intriguing differences in the importance of male vs. female age within pairs, which may relate to the relative costs of incubation and provisioning, and differences in provisioning rates between sexes and species. The drivers of ageing patterns in later life were more variable, with reproductive senescence and terminal effects only detectable in two of the three species, and selective disappearance contributing markedly to the ageing pattern in GHA. Our results show that even subtle differences in life histories and ecology of closely related species can be related to differences in the processes driving age‐related variation in breeding success in early and late adulthood. Some of these results are as predicted by life‐history theory, but others are not and may instead be related to ecological differences, such as in prey abundance and predation risk. Our work highlights the ecological importance of understanding among‐species differences in the processes driving population‐level patterns of ageing.

## AUTHORS’ CONTRIBUTIONS

H.F., S.L., D.H.N. and R.A.P. conceived and designed the study; R.A.P. oversees the long‐term albatross study; A.G.W. manages the long‐term data; H.F. conducted the analyses and drafted the manuscript; all authors contributed substantially to revisions and gave final approval for publication.

## DATA ACCESSIBILITY

Data available from the Dryad Digital Repository https://doi.org/10.5061/dryad.hm7k5 (Froy, Lewis, Nussey, Wood, & Phillips, 2017).

## References

[jane12712-bib-0001] ACAP. (2014a). Agreement on the conservation of Albatrosses and Petrels species assessment: Black‐browed Albatross Thalassarche melanophris. Retrieved from http://www.acap.aq(accessed april 2014).

[jane12712-bib-0002] ACAP. (2014b). Agreement on the conservation of Albatrosses and Petrels species assessment: Grey‐headed Albatross Thalassarche chrysostoma. Retrieved from http://www.acap.aq (accessed April 2014).

[jane12712-bib-0003] ACAP. (2014c). Agreement on the conservation of Albatrosses and Petrels species assessment: Wandering Albatross Diomedea exulans. Retrieved from http://www.acap.aq (accessed April 2014).

[jane12712-bib-0004] Aubry, L. M. , Koons, D. N. , Monnat, J.‐Y. , & Cam, E. (2009). Consequences of recruitment decisions and heterogeneity on age‐specific breeding success in a long‐lived seabird. Ecology, 90, 2491–2502. 19769127 10.1890/08-1475.1

[jane12712-bib-0005] Balbontín, J. , Møller, A. , Hermosell, I. , Marzal, A. , Reviriego, M. , & Lope, F. (2012). Geographical variation in reproductive ageing patterns and life‐history strategy of a short‐lived passerine bird. Journal of Evolutionary Biology, 25, 2298–2309. 22994532 10.1111/j.1420-9101.2012.02606.x

[jane12712-bib-0006] Barton, K. (2013). MuMIn: Multi‐model inference. R package version 1.15.6. Retrieved from https://CRAN.R-project.org/package=MuMIn.

[jane12712-bib-0007] Berman, M. , Gaillard, J.‐M. , & Weimerskirch, H. (2009). Contrasted patterns of age‐specific reproduction in long‐lived seabirds. Proceedings of the Royal Society B: Biological Sciences, 276, 375–382. 18832060 10.1098/rspb.2008.0925 PMC2674342

[jane12712-bib-0008] Berrow, S. D. , Humpidge, R. , & Croxall, J. P. (2000). Influence of adult breeding experience on growth and provisioning of Wandering Albatross *Diomedea exulans* chicks at South Georgia. Ibis, 142, 199–207.

[jane12712-bib-0009] Black, J. M. , & Hulme, M. F. (1996). Partnerships in birds: The study of monogamy. Oxford, UK: Oxford University Press.

[jane12712-bib-0010] Boonekamp, J. J. , Salomons, M. , Bouwhuis, S. , Dijkstra, C. , & Verhulst, S. (2014). Reproductive effort accelerates actuarial senescence in wild birds: An experimental study. Ecology Letters, 17, 599–605. 24818237 10.1111/ele.12263

[jane12712-bib-0011] Bouwhuis, S. , Charmantier, A. , Verhulst, S. , & Sheldon, B. C. (2010). Individual variation in rates of senescence: Natal origin effects and disposable soma in a wild bird population. Journal of Animal Ecology, 79, 1251–1261. 20646122 10.1111/j.1365-2656.2010.01730.x

[jane12712-bib-0012] Bouwhuis, S. , Sheldon, B. C. , Verhulst, S. , & Charmantier, A. (2009). Great tits growing old: Selective disappearance and the partitioning of senescence to stages within the breeding cycle. Proceedings of the Royal Society B: Biological Sciences, 276, 2769–2777. 19403537 10.1098/rspb.2009.0457 PMC2839957

[jane12712-bib-0013] Brunet‐Rossinni, A. K. , & Austad, S. N. (2006). Senescence in wild populations of mammals and birds In Masoro E. J., & Austad S. N. (Eds.), Handbook of the biology of aging (pp. 243–266). Burlington, MA: Elsevier Academic Press.

[jane12712-bib-0014] Burnham, K. P. , & Anderson, D. R. (2002). Model selection and multimodel inference: A practical information‐theoretic approach (2nd edn). London/New York: Springer.

[jane12712-bib-0015] Cam, E. , & Monnat, J.‐Y. (2000). Apparent inferiority of first‐time breeders in the kittiwake: The role of heterogeneity among age classes. Journal of Animal Ecology, 69, 380–394.

[jane12712-bib-0016] Clutton‐Brock, T. H. (1988). Reproductive success: Studies of individual variation in contrasting breeding systems. Chicago/London: University of Chicago Press.

[jane12712-bib-0017] Coulson, J. C. , & Fairweather, J. A. (2001). Reduced reproductive performance prior to death in the Black‐legged Kittiwake: Senescence or terminal illness? Journal of Avian Biology, 32, 146–152.

[jane12712-bib-0018] Croxall, J. P. , Prince, P. A. , Rothery, P. , & Wood, A. G. (1998). Population changes in albatrosses at South Georgia In Robertson G., & Gales R. (Eds.), Albatross biology and conservation (pp. 69–83). Chipping Norton, UK: Surrey Beatty.

[jane12712-bib-0019] Croxall, J. P. , Reid, K. , & Prince, P. A. (1999). Diet, provisioning and productivity responses of marine predators to differences in availability of Antarctic krill. Marine Ecology Progress Series, 177, 115–131.

[jane12712-bib-0020] Croxall, J. P. , & Ricketts, C. (1983). Energy costs of incubation in the Wandering Albatross *Diomedea exulans* . Ibis, 125, 33–39.

[jane12712-bib-0021] Croxall, J. P. , Rothery, P. , & Crisp, A. (1992). The effect of maternal age and experience on egg‐size and hatching success in Wandering Albatrosses *Diomedea exulans* . Ibis, 134, 219–228.

[jane12712-bib-0022] Croxall, J. P. , Rothery, P. , Pickering, S. P. C. , & Prince, P. A. (1990). Reproductive performance, recruitment and survival of wandering albatrosses *Diomedea exulans* at Bird Island, South Georgia. Journal of Animal Ecology, 59, 775–796.

[jane12712-bib-0023] Daunt, F. , Wanless, S. , Harris, M. P. , Money, L. , & Monaghan, P. (2007). Older and wiser: Improvements in breeding success are linked to better foraging performance in European shags. Functional Ecology, 21, 561–567.

[jane12712-bib-0024] Dugdale, H. L. , Pope, L. C. , Newman, C. , MacDonald, D. W. , & Burke, T. (2011). Age‐specific breeding success in a wild mammalian population: Selection, constraint, restraint and senescence. Molecular Ecology, 20, 3261–3274. 21714821 10.1111/j.1365-294X.2011.05167.x

[jane12712-bib-0025] Forslund, P. , & Pärt, T. (1995). Age and reproduction in birds — Hypotheses and tests. Trends in Ecology and Evolution, 10, 374–378. 21237076 10.1016/s0169-5347(00)89141-7

[jane12712-bib-0026] Forster, I. P. , & Phillips, R. A. (2009). Influence of nest location, density and topography on breeding success in the Black‐browed Albatross *Thalassarche melanophris* . Marine Ornithology, 37, 213–217.

[jane12712-bib-0027] Froy, H. , Lewis, S. , Nussey, D. H. , Wood, A. G. , & Phillips, R. A. (2017). Data from: Contrasting drivers of reproductive ageing in albatrosses. Dryad Digital Repository, https://doi.org/10.5061/dryad.hm7k5 10.1111/1365-2656.12712 PMC5601251 28605018

[jane12712-bib-0028] Froy, H. , Phillips, R. A. , Wood, A. G. , Nussey, D. H. , & Lewis, S. (2013). Age‐related variation in reproductive traits in the wandering albatross: Evidence for terminal improvement following senescence. Ecology Letters, 16, 642–649. 23438213 10.1111/ele.12092

[jane12712-bib-0029] Grueber, C. E. , Nakagawa, S. , Laws, R. J. , & Jamieson, I. G. (2011). Multimodel inference in ecology and evolution: Challenges and solutions. Journal of Evolutionary Biology, 24, 699–711. 21272107 10.1111/j.1420-9101.2010.02210.x

[jane12712-bib-0030] Hamel, S. , Gaillard, J.‐M. , Yoccoz, N. G. , Loison, A. , Bonenfant, C. , & Descamps, S. (2010). Fitness costs of reproduction depend on life speed: Empirical evidence from mammalian populations. Ecology Letters, 13, 915–935. 20482573 10.1111/j.1461-0248.2010.01478.x

[jane12712-bib-0031] Hammers, M. , Richardson, D. S. , Burke, T. , & Komdeur, J. (2012). Age‐dependent terminal declines in reproductive output in a wild bird. PLoS ONE, 7, e40413. 22792307 10.1371/journal.pone.0040413 PMC3391264

[jane12712-bib-0032] Hayward, A. D. , Mar, K. U. , Lahdenperä, M. , & Lummaa, V. (2014). Early reproductive investment, senescence and lifetime reproductive success in female Asian elephants. Journal of Evolutionary Biology, 27, 772–783. 24580655 10.1111/jeb.12350 PMC4237172

[jane12712-bib-0033] Hayward, A. D. , Wilson, A. J. , Pilkington, J. G. , Clutton‐Brock, T. H. , Pemberton, J. M. , & Kruuk, L. E. B. (2013). Reproductive senescence in female Soay sheep: Variation across traits and contributions of individual ageing and selective disappearance. Functional Ecology, 27, 184–195.

[jane12712-bib-0034] Holand, H. , Kvalnes, T. , Gamelon, M. , Tufto, J. , Jensen, H. , Pärn, H. , … Sæther, B.‐E. (2016). Spatial variation in senescence rates in a bird metapopulation. Oecologia, 181, 865–871. 27033720 10.1007/s00442-016-3615-4

[jane12712-bib-0035] Huin, N. , Prince, P. A. , & Briggs, D. R. (2000). Chick provisioning rates and growth in Black‐browed Albatross *Diomedea melanophris* and Grey‐headed Albatross *D. chrysostoma* at Bird Island, South Georgia. Ibis, 142, 550–565.

[jane12712-bib-0036] Jones, O. R. , Gaillard, J.‐M. , Tuljapurkar, S. , Alho, J. S. , Armitage, K. B. , Becker, P. H. , … Coulson, T. (2008). Senescence rates are determined by ranking on the fast–slow life‐history continuum. Ecology Letters, 11, 664–673. 18445028 10.1111/j.1461-0248.2008.01187.x

[jane12712-bib-0037] Loison, A. , Festa‐Bianchet, M. , Gaillard, J.‐M. , Jorgenson, J. T. , & Jullien, J.‐M. (1999). Age‐specific survival in five populations of ungulates: Evidence of senescence. Ecology, 80, 2539–2554.

[jane12712-bib-0038] Mazerolle, M. J. (2016). AICcmodavg: Model selection and multimodel inference based on (Q)AIC(c). R package version 2.1‐0. Retrieved from https://cran.r-project.org/package=AICcmodavg.

[jane12712-bib-0039] McCleery, R. H. , Perrins, C. M. , Sheldon, B. C. , & Charmantier, A. (2008). Age‐specific reproduction in a long‐lived species: The combined effects of senescence and individual quality. Proceedings of the Royal Society B: Biological Sciences, 275, 963–970. 18230597 10.1098/rspb.2007.1418 PMC2599932

[jane12712-bib-0040] Nevoux, M. , Forcada, J. , Barbraud, C. , Croxall, J. , & Weimerskirch, H. (2010). Bet‐hedging response to environmental variability, an intraspecific comparison. Ecology, 91, 2416–2427. 20836463 10.1890/09-0143.1

[jane12712-bib-0041] Nussey, D. H. , Coulson, T. , Delorme, D. , Clutton‐Brock, T. H. , Pemberton, J. M. , Festa‐Bianchet, M. , & Gaillard, J.‐M. (2011). Patterns of body mass senescence and selective disappearance differ among three species of free‐living ungulates. Ecology, 92, 1936–1947. 22073785 10.1890/11-0308.1

[jane12712-bib-0042] Nussey, D. H. , Coulson, T. , Festa‐Bianchet, M. , & Gaillard, J. M. (2008). Measuring senescence in wild animal populations: Towards a longitudinal approach. Functional Ecology, 22, 393–406.

[jane12712-bib-0043] Nussey, D. H. , Kruuk, L. E. B. , Donald, A. , Fowlie, M. , & Clutton‐Brock, T. H. (2006). The rate of senescence in maternal performance increases with early‐life fecundity in red deer. Ecology Letters, 9, 1342–1350. 17118008 10.1111/j.1461-0248.2006.00989.x

[jane12712-bib-0044] Pardo, D. , Barbraud, C. , Authier, M. , & Weimerskirch, H. (2013). Evidence for an age‐dependent influence of environmental variations on a long‐lived seabird's life‐history traits. Ecology, 94, 208–220. 23600255 10.1890/12-0215.1

[jane12712-bib-0045] Pardo, D. , Barbraud, C. , & Weimerskirch, H. (2013). Females better face senescence in the wandering albatross. Oecologia, 173, 1283–1294. 23797411 10.1007/s00442-013-2704-x

[jane12712-bib-0046] Péron, G. , Gimenez, O. , Charmantier, A. , Gaillard, J.‐M. , & Crochet, P.‐A. (2010). Age at the onset of senescence in birds and mammals is predicted by early‐life performance. Proceedings of the Royal Society B: Biological Sciences, 277, 2849–2856. 20427343 10.1098/rspb.2010.0530 PMC2981990

[jane12712-bib-0047] Phillips, R. A. , Gales, R. , Baker, G. B. , Double, M. C. , Favero, M. , Quintana, F. , … Wolfaardt, A. (2016). The conservation status and priorities for albatrosses and large petrels. Biological Conservation, 201, 169–183.

[jane12712-bib-0048] Phillips, R. A. , & Hamer, K. C. (2000). Growth and provisioning strategies of Northern Fulmars *Fulmarus glacialis* . Ibis, 142, 435–445.

[jane12712-bib-0049] Phillips, R. A. , Silk, J. R. D. , Phalan, B. , Catry, P. , & Croxall, J. P. (2004). Seasonal sexual segregation in two *Thalassarche albatross* species: Competitive exclusion, reproductive role specialization or foraging niche divergence? Proceedings of the Royal Society B: Biological Sciences, 271, 1283–1291. 15306353 10.1098/rspb.2004.2718 PMC1691717

[jane12712-bib-0050] Prince, P. A. , Rothery, P. , Croxall, J. P. , & Wood, A. G. (1994). Population‐dynamics of black‐browed and gray‐headed albatrosses *Diomedeamelanophris* and *D. chrysostoma* at bird island, South Georgia. Ibis, 136, 50–71.

[jane12712-bib-0051] Rattiste, K. (2004). Reproductive success in presenescent common gulls (*Larus canus*): The importance of the last year of life. Proceedings of the Royal Society B: Biological Sciences, 271, 2059–2064. 15451696 10.1098/rspb.2004.2832 PMC1691830

[jane12712-bib-0052] Rebke, M. , Coulson, T. , Becker, P. H. , & Vaupel, J. W. (2010). Reproductive improvement and senescence in a long‐lived bird. Proceedings of the National Academy of Sciences of the United States of America, 107, 7841–7846. 20378836 10.1073/pnas.1002645107 PMC2867923

[jane12712-bib-0053] Reed, T. E. , Kruuk, L. E. B. , Wanless, S. , Frederiksen, M. , Cunningham, E. J. A. , & Harris, M. P. (2008). Reproductive senescence in a long‐lived seabird: Rates of decline in late‐life performance are associated with varying costs of early reproduction. The American Naturalist, 171, E89–E101. 10.1086/524957 18173366

[jane12712-bib-0054] Reid, J. M. , Bignal, E. M. , Bignal, S. , McCracken, D. I. , Bogdanova, M. I. , & Monaghan, P. (2010). Parent age, lifespan and offspring survival: Structured variation in life history in a wild population. Journal of Animal Ecology, 79, 851–862. 20202008 10.1111/j.1365-2656.2010.01669.x

[jane12712-bib-0055] Reznick, D. , Nunney, L. , & Tessier, A. (2000). Big houses, big cars, superfleas and the costs of reproduction. Trends in Ecology and Evolution, 15, 421–425. 10998520 10.1016/s0169-5347(00)01941-8

[jane12712-bib-0056] Ricklefs, R. E. (2010). Life‐history connections to rates of aging in terrestrial vertebrates. Proceedings of the National Academy of Sciences of the United States of America, 107, 10314–10319. 20479246 10.1073/pnas.1005862107 PMC2890449

[jane12712-bib-0057] Robinson, M. R. , Mar, K. U. , & Lummaa, V. (2012). Senescence and age‐specific trade‐offs between reproduction and survival in female Asian elephants. Ecology Letters, 15, 260–266. 22248152 10.1111/j.1461-0248.2011.01735.x

[jane12712-bib-0058] Rose, M. R. (1991). Evolutionary biology of aging. New York, NY: Oxford University Press.

[jane12712-bib-0059] Ryan, P. G. , Phillips, R. A. , Nel, D. C. , & Wood, A. G. (2007). Breeding frequency in grey‐headed Albatrosses *Thalassarche chrysostoma* . Ibis, 149, 45–52.

[jane12712-bib-0060] Stearns, S. C. (1992). The evolution of life histories. Oxford; New York: Oxford University Press.

[jane12712-bib-0061] Ulm, K. , & Cox, C. (1989). On the estimation of threshold values. Biometrics, 45, 1324–1328.

[jane12712-bib-0062] van de Pol, M. , & Verhulst, S. (2006). Age‐dependent traits: A new statistical model to separate within‐ and between‐individual effects. American Naturalist, 167, 766–773. 10.1086/503331 16671020

[jane12712-bib-0063] Vaupel, J. W. , Manton, K. G. , & Stallard, E. (1979). The impact of heterogeneity in individual frailty on the dynamics of mortality. Demography, 16, 439–454. 510638

[jane12712-bib-0064] Weimerskirch, H. (1992). Reproductive effort in long‐lived birds: Age‐specific patterns of condition, reproduction and survival in the wandering albatross. Oikos, 64, 464–473.

[jane12712-bib-0065] Weimerskirch, H. (1995). Regulation of foraging trips and incubation routine in male and female wandering albatrosses. Oecologia, 102, 37–43. 28306805 10.1007/BF00333308

[jane12712-bib-0066] Williams, G. C. (1966). Natural selection, the costs of reproduction, and a refinement of Lack's Principle. The American Naturalist, 100, 687–690.

[jane12712-bib-0067] Xavier, J. C. , Croxall, J. P. , & Reid, K. (2003). Interannual variation in the diets of two albatross species breeding at South Georgia: Implications for breeding performance. Ibis, 145, 593–610.

[jane12712-bib-0068] Xavier, J. C. , Trathan, P. N. , Croxall, J. P. , Wood, A. G. , Podesta, G. , & Rodhouse, P. G. (2004). Foraging ecology and interactions with fisheries of wandering albatrosses (*Diomedea exulans*) breeding at South Georgia. Fisheries Oceanography, 13, 324–344.

[jane12712-bib-0069] Zhang, H. , Vedder, O. , Becker, P. H. , & Bouwhuis, S. (2015). Age‐dependent trait variation: The relative contribution of within‐individual change, selective appearance and disappearance in a long‐lived seabird. Journal of Animal Ecology, 84, 797–807. 25399484 10.1111/1365-2656.12321

[jane12712-bib-0070] Zuur, A. F. , Ieno, E. N. , Walker, N. , Saveliev, A. A. , & Smith, G. M. (2009). Mixed effects models and extensions in ecology with R. New York; London: Springer.

