# Supplementary material for: Contrasting drivers of reproductive ageing in albatrosses
Source: J Anim Ecol. 2017 Jul 17;86(5):1022–32. doi: 10.1111/1365-2656.12712 (PMC5601251; doi:10.1111/1365-2656.12712)
Supplement: Supplementary file 1 [file JANE-86-1022-s001.pdf]

Supplementary material 2: Supplementary tables

Table S1. Models describing the relationship between age and breeding success in three species of albatrosses breeding at Bird Island, South Georgia. Tables show null models (ID: random intercept for individual; Year + ID: random intercepts for year and individual; Age: fixed linear covariate for age plus random intercepts for year and individual), and the top model set (where ΔAIC<2 compared to the model with the lowest AIC score, see Methods), ordered by AIC. Numbers shown in the Model column indicate the threshold values fitted.

Black-browed albatross

Males			Females					
Model	AIC	ΔAIC	Model	AIC	ΔAIC	Model	AIC	ΔAIC
18	2760.04	0.00	23	1850.44	0.00	24 26	1852.28	1.84
15 18	2760.40	0.37	22	1850.63	0.19	23 26	1852.30	1.86
19	2760.53	0.50	21	1851.01	0.57	21 28	1852.31	1.88
20	2760.91	0.87	Age^2	1851.05	0.61	20 29	1852.32	1.88
14 18	2760.95	0.92	24	1851.08	0.64	21 25	1852.35	1.91
16 18	2760.97	0.93	20	1851.27	0.84	20 26	1852.35	1.91
13 18	2761.50	1.47	20 24	1851.81	1.37	20 27	1852.37	1.93
18 25	2761.59	1.55	13 23	1851.82	1.39	25	1852.37	1.94
12 18	2761.73	1.69	15 23	1851.84	1.40	23 27	1852.38	1.94
18 26	2761.77	1.73	19 24	1851.84	1.40	22 28	1852.38	1.95
18 24	2761.78	1.74	14 23	1851.87	1.43	23 25	1852.41	1.97
18 27	2761.84	1.80	18 23	1851.87	1.43	22 24	1852.42	1.98
17	2761.86	1.82	18 24	1851.89	1.45	23 28	1852.42	1.99
18 23	2761.86	1.82	19 23	1851.89	1.45	23 32	1852.43	2.00
18 28	2761.93	1.89	20 23	1851.90	1.47	23 29	1852.44	2.00
18 21	2761.94	1.90	17 23	1851.92	1.48	23 30	1852.44	2.00
18 20	2761.95	1.92	20 25	1851.94	1.50	23 31	1852.44	2.00
18 29	2761.98	1.94	20 28	1852.01	1.57	Age	1867.80	17.36
21	2761.98	1.94	16 23	1852.01	1.57	Year+ID	1870.61	20.17
18 30	2761.99	1.95	17 24	1852.02	1.59	ID	1962.35	111.91
18 22	2762.01	1.97	15 24	1852.06	1.63			
18 31	2762.03	1.99	19 25	1852.09	1.65			
18 33	2762.04	2.00	14 24	1852.18	1.74			
11 18	2762.04	2.00	13 24	1852.19	1.75			
18 32	2762.04	2.00	16 24	1852.22	1.78			
Age	2777.98	17.94	21 24	1852.23	1.79			
Year+ID	2793.31	33.27	21 23	1852.24	1.81			
ID	2993.22	233.18	18 25	1852.26	1.82			

Grey-headed albatross								
Males			Females					
Model	AIC	ΔAIC	Model	AIC	ΔAIC	Model	AIC	ΔAIC
24 27	2718.56	0.00	26	2680.61	0.00	26 33	2682.50	1.89
22	2718.78	0.23	25	2680.64	0.03	26 29	2682.51	1.90
23 27	2718.93	0.37	26 43	2680.66	0.05	26 28	2682.51	1.90
24 26	2719.14	0.58	27	2680.74	0.13	26 30	2682.53	1.92
23	2719.39	0.84	21 27	2680.83	0.22	24 30	2682.53	1.92
21	2719.46	0.90	28 43	2680.94	0.33	20 29	2682.55	1.94
23 32	2719.47	0.92	21 28	2681.05	0.44	26 32	2682.56	1.95
22 32	2719.51	0.96	22 27	2681.21	0.60	23 34	2682.57	1.96
22 33	2719.53	0.98	25 43	2681.26	0.65	26 37	2682.57	1.96
23 33	2719.55	0.99	20 27	2681.40	0.79	26 41	2682.58	1.97
22 27	2719.56	1.01	22 28	2681.40	0.79	26 31	2682.59	1.98
23 26	2719.57	1.01	21 29	2681.44	0.83	26 40	2682.60	1.99
22 41	2719.73	1.18	23 27	2681.49	0.88	26 39	2682.60	1.99
22 34	2719.80	1.24	23 28	2681.62	1.01	26 38	2682.60	1.99
22 31	2719.87	1.31	22 29	2681.71	1.10	Year+ID	2740.59	59.98
22 40	2719.87	1.32	23 29	2681.83	1.21	Age	2741.50	60.88
22 42	2719.89	1.33	20 28	2681.88	1.26	ID	2996.82	316.21
23 31	2719.90	1.34	25 34	2681.94	1.33			
23 28	2719.91	1.35	24 27	2681.98	1.37			
23 34	2719.93	1.38	26 42	2681.99	1.37			
23 29	2719.95	1.39	25 27	2681.99	1.38			
22 29	2720.01	1.45	25 35	2682.00	1.39			
23 41	2720.01	1.45	25 28	2682.01	1.40			
22 28	2720.04	1.49	25 29	2682.02	1.41			
22 26	2720.06	1.50	25 30	2682.10	1.49			
23 25	2720.09	1.53	25 36	2682.11	1.50			
22 30	2720.12	1.56	24 28	2682.12	1.51			
25 27	2720.13	1.57	25 33	2682.14	1.52			
23 40	2720.16	1.60	21 30	2682.14	1.52			
22 35	2720.16	1.61	23 30	2682.21	1.60			
22 39	2720.16	1.61	25 32	2682.24	1.63			
24 32	2720.16	1.61	24 29	2682.27	1.66			
23 30	2720.20	1.64	22 30	2682.27	1.66			
23 42	2720.22	1.66	25 31	2682.30	1.69			
24 33	2720.30	1.74	28	2682.32	1.71			
22 38	2720.32	1.76	24	2682.34	1.73			
22 36	2720.36	1.80	25 42	2682.34	1.73			
24 28	2720.37	1.82	19 27	2682.36	1.75			
22 37	2720.37	1.82	25 37	2682.37	1.76			
22 25	2720.40	1.85	26 34	2682.38	1.77			
23 35	2720.42	1.87	26 35	2682.40	1.79			
23 39	2720.49	1.93	29 43	2682.40	1.79			
24 29	2720.54	1.99	26 36	2682.45	1.84			
Year+ID	2734.61	16.05	25 38	2682.47	1.86			
Age	2736.43	17.88	25 39	2682.48	1.87			
ID	3058.22	339.66	25 40	2682.50	1.89			

Wandering albatross

Males						Females		
Model	AIC	ΔAIC	Model	AIC	ΔAIC	Model	AIC	ΔAIC
18	2841.96	0.00	18 22	2843.64	1.68	14	2837.06	0.00
20	2842.04	0.07	20 23	2843.65	1.68	15	2837.69	0.64
12 20	2842.16	0.20	14 22	2843.66	1.70	15 26	2838.63	1.57
19	2842.18	0.21	12 25	2843.67	1.71	15 27	2838.66	1.60
12 21	2842.40	0.44	12 26	2843.69	1.72	14 27	2838.71	1.65
12 22	2842.59	0.63	18 21	2843.70	1.73	14 26	2838.73	1.67
Age^2	2842.86	0.89	12 23	2843.72	1.75	14 16	2838.78	1.72
21	2843.06	1.10	16 20	2843.74	1.78	14 17	2838.80	1.75
15 20	2843.08	1.12	13 22	2843.76	1.80	14 28	2838.84	1.79
13 20	2843.18	1.22	18 35	2843.76	1.80	14 25	2838.91	1.85
15 27	2843.20	1.23	18 24	2843.80	1.83	15 25	2838.93	1.88
15 21	2843.21	1.24	19 27	2843.80	1.84	14 18	2838.94	1.88
18 27	2843.22	1.25	19 29	2843.82	1.85	15 28	2838.96	1.91
15 22	2843.22	1.26	17 20	2843.83	1.86	14 29	2838.98	1.93
14 20	2843.25	1.29	19 28	2843.84	1.88	14 19	2839.00	1.94
18 29	2843.28	1.31	20 24	2843.84	1.88	14 24	2839.02	1.97
18 28	2843.28	1.32	18 23	2843.84	1.88	14 23	2839.05	1.99
15 26	2843.41	1.45	17 27	2843.87	1.91	14 20	2839.05	1.99
18 26	2843.42	1.45	19 31	2843.90	1.93	14 34	2839.05	2.00
18 31	2843.42	1.46	15 23	2843.90	1.93	14 30	2839.05	2.00
18 30	2843.45	1.48	20 22	2843.91	1.95	14 31	2839.06	2.00
15 28	2843.47	1.51	20 29	2843.93	1.97	14 22	2839.06	2.00
17	2843.48	1.51	19 30	2843.93	1.97	14 33	2839.06	2.00
18 33	2843.50	1.54	19 33	2843.93	1.97	14 21	2839.06	2.00
14 21	2843.52	1.55	21 23	2843.94	1.98	14 35	2839.06	2.00
13 21	2843.52	1.56	15 24	2843.94	1.98	Year+ID	2881.80	44.74
15 25	2843.55	1.58	20 27	2843.95	1.99	Age	2883.26	46.20
18 25	2843.56	1.60	20 31	2843.95	1.99	ID	2951.97	114.91
18 32	2843.59	1.62	20 28	2843.96	1.99			
12 27	2843.60	1.63	20 33	2843.96	1.99			
15 29	2843.61	1.64	Year+ID	2862.23	20.27			
18 34	2843.63	1.66	Age	2862.60	20.64			
18 20	2843.63	1.67	ID	2920.69	78.73			

Table S2. The best single threshold models describing the relationship between age and breeding success, estimated using GLMMs with year and bird ID as random effects. The best threshold is shown in bold, with 95% confidence intervals. The estimates, standard error (S.E.) and significance of the regression slopes before and after the threshold break point are indicated.

			Threshold			Before threshold			After threshold		
		n	age	lower CI	upper CI	Estimate	S.E.	p-value	Estimate	S.E.	p-value
BBA	Males	2273	**18**	16.6	21.4	0.113	0.020	<0.001	-0.011	0.013	0.410
	Females	1521	**23**	18.7	25.3	0.104	0.022	<0.001	-0.059	0.023	0.010
GHA	Males	2267	**22**	19.5	24.4	0.093	0.023	<0.001	-0.049	0.013	<0.001
	Females	2225	**26**	23.2	28.4	0.106	0.017	<0.001	-0.130	0.018	<0.001
WA	Males	2255	**18**	14.9	22.2	0.095	0.019	<0.001	-0.041	0.013	0.001
	Females	2260	**14**	13.5	16.2	0.210	0.031	<0.001	-0.040	0.009	<0.001

Table S3. Models of breeding success estimated using GLMMs with year, male ID and female ID included as random effects. For each species, the best single threshold age functions for both males and females were included (T1). The effect of removing the male or female age term on the model AIC is shown (ΔAIC). The estimates, standard error (S.E.) and significance of the regression slopes before and after the threshold break point are indicated for both the male and female age functions. Statistically significant terms are highlighted in bold.

		Male age term						Female age term					
	n	T1	ΔAIC		Estimate	S.E.	p-value	T1	ΔAIC		Estimate	S.E.	p-value
BBA	944	**18**	**7.271**	**before T1**	**0.117**	**0.036**	**0.001**	23	-1.638	before T1	0.051	0.030	0.097
				after T1	-0.014	0.034	0.679			after T1	-0.047	0.040	0.242
GHA	881	22	-2.680	before T1	0.034	0.045	0.443	**26**	**11.394**	**before T1**	**0.099**	**0.035**	**0.005**
				after T1	-0.026	0.026	0.328			**after T1**	**-0.145**	**0.041**	**<0.001**
WA	1561	18	-3.357	before T1	0.020	0.034	0.554	**14**	**16.597**	**before T1**	**0.212**	**0.049**	**<0.001**
				after T1	-0.15	0.021	0.487			**after T1**	**-0.043**	**0.018**	**0.018**

Table S4. Estimated fixed effects (estimate (Est.) and standard error (S.E.)) from GLMMs of breeding success for a) males; and b) females during early adulthood. Data for all three species were included in a single model for each sex, and the interaction between species and each of the three age terms tested. Both models included year and bird ID as random effects. Estimates for the main effects are for BBA, and the interaction terms show the effects for GHA and WA relative to BBA. The effect of removing each interaction on the model AIC is shown (ΔAIC). Positive ΔAIC values indicate that the term improved model fit, and those interactions whose removal increased AIC by >2 are highlighted in bold.

		a) Males n=2823			b) Females n=2585		
		Est.	S.E.	ΔAIC	Est.	S.E.	ΔAIC
Main effects	Species: GHA	0.736	0.971		1.798	1.036	
	Species: WA	1.880	0.818		2.505	0.837	
	Years since first bred	0.172	0.037		0.170	0.035	
	Age at first repro	-0.046	0.065		0.097	0.052	
	First breeding attempt	-0.042	0.269		-0.495	0.351	
Interactions	Years since first bred * Species GHA	**-0.126**	**0.055**	**5.769**	**-0.126**	**0.045**	**4.778**
	Years since first bred * Species WA	**-0.148**	**0.050**		**-0.040**	**0.049**	
	Age at first repro * Species GHA	0.019	0.083	-3.228	-0.102	0.077	-2.188
	Age at first repro * Species WA	0.063	0.080		-0.034	0.071	
	First breeding attempt * Species GHA	-0.326	0.397	1.613	-0.217	0.462	-3.210
	First breeding attempt * Species WA	-0.774	0.334		0.099	0.398	

Table S5. Estimated fixed effects (estimate (Est.) and standard error (S.E.)) from GLMMs of breeding success for a) males; and b) females during late adulthood. Data for all three species were included in a single model for each sex, and the interaction between species and each of the three age terms tested. Both models included year and bird ID as random effects. Estimates for the main effects are for BBA, and the interaction terms show the effects for GHA and WA relative to BBA. The effect of removing each interaction on the model AIC is shown (ΔAIC). Positive ΔAIC values indicate that the term improved model fit, and those interactions whose removal increased AIC by >2 are highlighted in bold.

		a) Males n=2004			b) Females n=1387		
		Est.	S.E.	ΔAIC	Est.	S.E.	ΔAIC
Main effects	Species: GHA	0.974	0.895		-0.773	1.721	
	Species: WA	0.599	0.806		-2.453	1.577	
	Years before death	0.078	0.033		0.087	0.061	
	Age at last repro	-0.052	0.025		-0.144	0.050	
	Last breeding attempt	-0.090	0.332		0.069	0.539	
Interactions	Years before death * Species GHA	0.014	0.037	-3.834	**0.096**	**0.065**	**4.315**
	Years before death * Species WA	0.007	0.040		**-0.013**	**0.066**	
	Age at last repro * Species GHA	-0.016	0.030	-1.593	**0.035**	**0.055**	**4.896**
	Age at last repro * Species WA	0.021	0.029		**0.116**	**0.053**	
	Last breeding attempt * Species GHA	0.846	0.412	0.405	0.708	0.617	-2.479
	Last breeding attempt * Species WA	0.667	0.404		0.707	0.602	

Table S6. Estimated fixed effects (estimate (Est.) and standard error (S.E.)) from GLMMs of breeding success for birds during late adulthood, accounting for the effects of partner change. All models included year and bird ID as random effects. The effect of removing each parameter on the model AIC is shown (ΔAIC). Positive ΔAIC values indicate that the term improved model fit, and those parameters whose removal increased AIC by >2 are highlighted in bold.

			Years before death			Age at last repro			Last breeding attempt			Partner change		
		n	Est.	S.E.	ΔAIC	Est.	S.E.	ΔAIC	Est.	S.E.	ΔAIC	Est.	S.E.	ΔAIC
BBA	Males	441	0.069	0.036	1.672	-0.030	0.023	-0.241	-0.091	0.348	-1.933	-0.204	0.321	-1.598
	Females	172	0.030	0.028	-1.828	-0.109	0.063	0.940	-0.060	0.618	-1.990	-0.894	0.690	-0.150
GHA	Males	817	**0.093**	**0.028**	**9.287**	**-0.065**	**0.024**	**5.369**	**0.828**	**0.274**	**7.014**	-0.312	0.254	-0.506
	Females	570	**0.204**	**0.049**	**17.084**	**-0.108**	**0.042**	**4.802**	**0.991**	**0.374**	**5.154**	0.301	0.333	-1.185
WA	Males	711	**0.077**	**0.026**	**7.286**	-0.031	0.017	1.257	**0.540**	**0.235**	**3.308**	-0.137	0.354	-1.850
	Females	575	**0.068**	**0.026**	**5.210**	-0.025	0.017	-0.047	**0.695**	**0.260**	**5.272**	-0.581	0.301	1.796

Table S7. Estimated fixed effects (estimate (Est.) and standard error (S.E.)) from GLMMs of breeding success for birds during late adulthood, accounting for the number of years since the bird last bred, the outcome of the previous breeding attempt, and the interaction between these terms. All models included year and bird ID as random effects. The effect of removing each parameter on the model AIC is shown (ΔAIC). Positive ΔAIC values indicate that the term improved model fit, and those parameters whose removal increased AIC by >2 are highlighted in bold.

			Years before death			Age at last repro			Last breeding attempt		
		n	Est.	S.E.	ΔAIC	Est.	S.E.	ΔAIC	Est.	S.E.	ΔAIC
BBA	Males	436	0.067	0.037	1.252	-0.025	0.023	-0.839	-0.214	0.359	-1.648
	Females	169	0.007	0.073	-1.992	-0.088	0.064	-0.230	-0.238	0.638	-1.859
GHA	Males	812	**0.086**	**0.028**	**7.447**	**-0.062**	**0.024**	**4.789**	**0.809**	**0.274**	**6.628**
	Females	570	**0.189**	**0.048**	**14.812**	**-0.100**	**0.041**	**4.156**	**1.005**	**0.368**	**5.610**
WA	Males	594	**0.086**	**0.028**	**7.878**	**-0.033**	**0.015**	**2.479**	**0.564**	**0.251**	**3.071**
	Females	498	0.054	0.028	1.923	-0.029	0.017	0.848	**0.754**	**0.276**	**5.607**
